# When Unilateral Leg Swelling Is Not Deep Vein Thrombosis: A Case of Disseminated Kaposi Sarcoma Revealing Advanced Human Immunodeficiency Virus

**DOI:** 10.7759/cureus.109176

**Published:** 2026-05-19

**Authors:** Ahmed Ismail, Zachary Wilson, Thomas Curow, Michael DiLeonardo, Carsten Kirby

**Affiliations:** 1 Department of Medicine, Eastern Virginia Medical School at Old Dominion University, Norfolk, USA

**Keywords:** hhv‑8, hiv/aids, kaposi sarcoma, lymphadenopathy, ocular syphilis, pulmonary nodules, unilateral leg edema

## Abstract

Kaposi sarcoma (KS) is an acquired immunodeficiency syndrome (AIDS)-defining malignancy that can involve the skin, lymph nodes, and visceral organs in patients with advanced human immunodeficiency virus (HIV). We report the case of a 35-year-old man with no known prior medical history who presented to the charity care clinic complaining of recurrent thrush and two weeks of progressive right lower extremity swelling. His physical examination revealed painful, pruritic violaceous papules and plaques over the proximal right thigh that had been present for one week. He was referred to the emergency department (ED) for further workup. Venous duplex ultrasonography was negative for deep venous thrombosis (DVT). He was found to have a newly diagnosed HIV-1 infection with advanced immunosuppression, oral thrush with odynophagia and dysphagia concerning for esophageal candidiasis, and a reactive rapid plasma reagin titer of 1:32 with retinal white dot lesions concerning for ocular syphilis. Computed tomography (CT) demonstrated diffuse right lower extremity soft tissue edema, extensive right inguinal, external iliac, and intrapelvic lymphadenopathy, bilateral axillary lymphadenopathy, and multiple bilateral pulmonary nodules. Punch biopsy of a right thigh lesion revealed KS with tumor cells positive for cluster of differentiation 31 (CD31), CD34, ERG, and human herpesvirus 8 (HHV-8), and an excisional lymph node biopsy demonstrated findings consistent with disseminated KS. Furthermore, cerebrospinal fluid (CSF) analysis confirmed neurosyphilis with a positive Venereal Disease Research Laboratory (VDRL). He was treated with bictegravir/emtricitabine/tenofovir alafenamide, trimethoprim-sulfamethoxazole prophylaxis, fluconazole, and intravenous penicillin G. The patient showed early clinical improvement with resolution of ocular symptoms and a declining HIV viral load following initiation of therapy and before discharge.

This case highlights biopsy-proven KS as the presenting manifestation of previously undiagnosed advanced HIV, with unilateral limb lymphedema and pulmonary nodules suggesting disseminated disease. Additionally, our case underscores the need for early HIV testing in patients with recurrent mucocutaneous candidiasis and unexplained lymphedema, and for prompt evaluation of concomitant opportunistic and sexually transmitted infections.

## Introduction

Kaposi sarcoma (KS) is an angioproliferative neoplasm driven by infection with human herpesvirus 8 (HHV-8) and remains an acquired immunodeficiency syndrome (AIDS)-defining malignancy in individuals with human immunodeficiency virus (HIV) infection [1]. It is characterized by abnormal vascular proliferation and may involve the skin, lymph nodes, mucosal surfaces, and visceral organs, including the lungs and gastrointestinal tract [1]. Although the incidence of KS has declined in the era of antiretroviral therapy (ART), it continues to occur in patients with delayed HIV diagnosis or advanced immunosuppression, where it may present with atypical clinical features that mimic infectious, vascular, or malignant conditions [1-3]. In high‑income settings, KS has become relatively uncommon in the general population, with an estimated incidence of only a few cases per million persons per year, but it remains markedly more frequent among people living with HIV despite widespread ART use [4]. This decline compared with the pre‑ART era underscores that contemporary cases often arise in the context of delayed diagnosis, treatment interruptions, or advanced immunosuppression, as in our patient.

Cutaneous KS classically presents as violaceous macules, plaques, or nodules; however, presentations such as unilateral limb edema due to lymphatic or nodal obstruction can obscure the diagnosis and delay appropriate evaluation. Early recognition is critical, as prompt initiation of ART is the cornerstone of management and may lead to regression in limited disease. At the same time, more advanced or symptomatic cases may require systemic therapy in addition to ART [1-4].

In patients with advanced HIV, concurrent opportunistic and sexually transmitted infections must also be considered. Ocular syphilis is a vision-threatening manifestation of syphilis that may occur at any stage and requires urgent evaluation. According to Centers for Disease Control and Prevention guidelines, patients with ocular symptoms and reactive syphilis serology should undergo prompt ophthalmologic assessment, and suspected ocular syphilis should be treated using neurosyphilis regimens with intravenous penicillin G for 10-14 days [5].

Here, we report a case of newly diagnosed advanced HIV infection presenting with unilateral right lower extremity swelling and biopsy-proven KS, accompanied by generalized lymphadenopathy, pulmonary nodules suspicious for disseminated involvement, esophageal candidiasis, and ocular neurosyphilis.

## Case presentation

**Figure 1:**
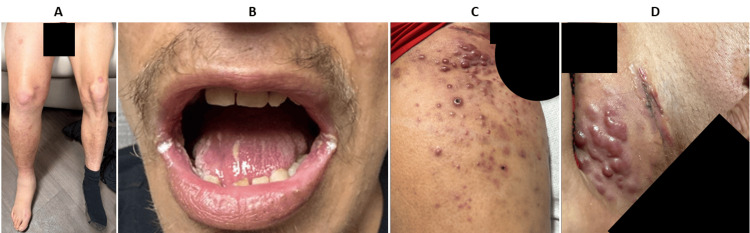
Cutaneous and Mucosal Manifestations on Presentation The clinical photographs demonstrate cutaneous Kaposi sarcoma and concomitant mucocutaneous candidiasis in a patient with advanced HIV. (A) Asymmetric right lower extremity swelling. (B) Oral cavity showing pseudomembranous white plaques along the tongue and buccal mucosa consistent with oral candidiasis. (C) Clustered violaceous papules and nodules over the right proximal thigh. (D) Confluent violaceous nodules and plaques in the right inguinal region following excisional lymph node biopsy. HIV: human immunodeficiency virus

A 35-year-old man with a past medical history significant solely for oral thrush, which was treated with fluconazole monotherapy without much improvement, presented from the charity care clinic to the emergency department (ED) with progressive swelling of the right lower extremity for approximately two weeks and a painful, pruritic rash over the right proximal thigh for one week (Figure 1). The lesions began as scaly spots and became raised, firm, violaceous papules and plaques. Some lesions bled when scratched. The onset of the lesions and swelling was accompanied by malaise, fatigue, chronic non-productive cough, sore throat with odynophagia/dysphagia, and photophobia. No fever, chills, night sweats, dyspnea, abdominal pain, diarrhea, dysuria, urethral discharge, headaches, dizziness, focal weakness, or significant weight loss were reported. Social history was notable for sex with men, with the last reported sexual activity approximately five years earlier. Around that time, he had tested negative for HIV but positive for syphilis and received intramuscular penicillin treatment through the health department.

On presentation, he was afebrile and hemodynamically stable. Physical examination showed marked right leg edema and violaceous, firm papules and plaques over the right proximal anterior and medial thigh, with right inguinal lymphadenopathy (Figure 1). Oral examination showed diffuse white plaques concerning for thrush (Figure 1).

Initial laboratory studies

Initial laboratory studies revealed mild anemia (hemoglobin: 11.4 g/dL), borderline macrocytosis (mean corpuscular volume (MCV): 97 fL), and mild transaminitis (aspartate transaminase (AST): 55 U/L, alanine aminotransferase (ALT): 41 U/L). Hepatitis testing (A, B, and C) was non-reactive. Serum studies demonstrated hyperproteinemia with elevated globulin (4.7 g/dL) and a low albumin-to-globulin ratio (0.8), consistent with hypergammaglobulinemia. HIV screening was reactive, with confirmatory HIV-1 antibody positivity. Further evaluation showed a cluster of differentiation 4 (CD4) count of 72 cells/µL (4%) and an HIV-1 RNA of 301,000 copies/mL (log10 5.48), consistent with advanced immunosuppression. Rapid plasma reagin was reactive at 1:32 with positive treponemal IgG/IgM (pertinent laboratory studies are summarized in Table 1).

**Table 1:** Pertinent Laboratory Studies Overall, the laboratory profile is consistent with advanced, uncontrolled HIV infection (severe CD4 lymphopenia and high HIV-1 RNA) and chronic immune activation, with evidence of neurosyphilis and mucocutaneous candidiasis. WBC: white blood cell count, MCV: mean corpuscular volume, CD4: cluster of differentiation 4, SPEP: serum protein electrophoresis, AST: aspartate aminotransferase, ALT: alanine aminotransferase, ALP: alkaline phosphatase, CPK: creatine phosphokinase, A/G ratio: albumin-to-globulin ratio, HIV: human immunodeficiency virus, Ab/Ag: antibody/antigen, RPR: rapid plasma reagin, IgM: immunoglobulin M, IgG: immunoglobulin G, TB: tuberculosis, GC: Neisseria gonorrhoeae, cm H₂O: centimeters of water, IU/mL: international units per milliliter, g/dL: grams per deciliter, K/µL: thousand cells per microliter, cells/µL: cells per microliter, U/L: units per liter, ng/mL: nanograms per milliliter, pg/mL: picograms per milliliter, RBC: red blood cells, ART: antiretroviral therapy, VDRL: Venereal Disease Research Laboratory, CSF: cerebrospinal fluid

Test	Result	Reference range
Pertinent hematologic workup		
Hemoglobin	11.4 g/dL	13.2-17.3 g/dL
MCV	97 fL	80-95 fL
WBC	4.9 K/uL	4-11 K/µL
Platelets	245 K/uL	140-440 K/µL
CD4 count	72 cells/µL	500-1,500 cells/µL
CD4 %	4%	30%-60%
SPEP		
Total protein	7.7 g/dL	6.4-8.3 g/dL
Albumin	37.9%	46.6%-62.6%
Alpha-1 globulin	3.2%	1.7%-4.1%
Alpha-2 globulin	8.7%	5.9%-13.5%
Beta globulin	10.3%	10.9%-18.9%
Gamma globulin	39.9%	11.6%-24.4%
Pertinent chemistry workup		
AST	55 U/L	10-37 U/L
ALT	41 U/L	5-40 U/L
ALP	90 U/L	25-115 U/L
Total bilirubin	0.5	0.2-1.2 mg/dL
Total protein	8.4 g/dL	6.0-8.3 g/dL
Albumin	3.7 g/dL	3.5-5.0 g/dL
Globulin	4.7 g/dL	2.0-4.0 g/dL
A/G ratio	0.8	1.0-2.0
CPK	224	30-200 U/L
Folate	9.10 ng/mL	*≥*3.10 ng/mL
Vitamin B12	559 pg/mL	211-911 pg/mL
Pertinent infectious workup		
HIV-1/0/2 Ab/Ag screening	Reactive	Non-reactive
HIV-1 antibody	Reactive	Non-reactive
HIV-2 antibody	Non-reactive	Non-reactive
HIV RNA	301,000 copies/mL	Undetectable
HIV RNA (8 days after initiating ART)	5,440 copies/mL	Undetectable
RPR	Reactive	Non-reactive
RPR titer	1:32	<1:1
Syphilis IgM/IgG	Reactive	Non-reactive
QuantiFERON TB Gold Plus	Negative	Negative
QuantiFERON nil value	0.14 IU/mL	-
QuantiFERON TB1 Ag	0.04 IU/mL	-
QuantiFERON TB2 Ag value	0.02 IU/mL	-
Hepatitis A IgM	Non-detected	Non-detected
Hepatitis B core IgM	Non-detected	Non-detected
Hepatitis B surface Ag	Non-detected	Non-detected
Hepatitis C Ab	Non-detected	Non-detected
Cryptococcal antigen	Negative	Negative
Chlamydia amplified urine	Negative	Negative
GC amplified urine	Negative	Negative
Trichomonas Nuc amplified urine	Negative	Negative
Pertinent CSF studies		
Opening pressure	22 cm H₂O	10- 20 cm H₂O
Appearance	Clear, colorless	Clear, colorless
WBC	22	0-5 cells/µL
RBC	45	0-5 cells/µL
Neutrophils	0%	0%-6%
Lymphocytes	97%	40%-80%
Monocytes	3%	15%-45%
Protein	94 mg/dL	12-60 mg/dL
Glucose	49 mg/dL	40-70 mg/dL
VDRL	1:2	Non-reactive
HIV	Non-detected	Non-detected

These results indicated profound cellular immunosuppression, with a low CD4 count and a high HIV‑1 RNA level consistent with advanced HIV disease. The hypergammaglobulinemia and low albumin‑to‑globulin ratio further supported chronic immune activation in the setting of long-standing, untreated infection.

Initial imaging studies

**Figure 2:**
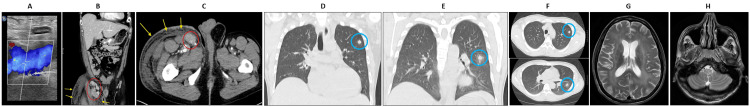
Pertinent Imaging Findings (A) Representative image of the duplex ultrasound of the right lower extremity showing no deep venous thrombosis. (B and C) Representative images of the coronal and axial CT of the pelvis, respectively, showing an enlarged right inguinal lymph node (red circle) and soft tissue edema (yellow arrows). (D and E) Representative images of the coronal CT of the chest showing pulmonary nodules (blue circle). (F) Representative images of the axial CT of the chest showing pulmonary nodules (blue circles). (G and H) Representative image of the MRI of the brain showing diffuse abnormal signal intensity of the cerebrum in G and the brain stem in H. CT: computed tomography, MRI: magnetic resonance imaging

Venous duplex ultrasonography of the right lower extremity showed no evidence of deep venous thrombosis (DVT) (Figure 2). Computed tomography (CT) of the abdomen and pelvis revealed enlarged right greater than left pelvic, external iliac, and inguinal lymph nodes, including a 2.5 cm right inguinal node with abnormal morphology and heterogeneous enhancement (Figure 2). CT of the right lower extremity demonstrated moderate anterolateral and mild circumferential soft tissue edema extending from the hip to the dorsal foot, with nodular subcutaneous plaques over the medial proximal thigh and probable lymphadenopathy along the distal thigh and popliteal neurovascular chain. CT of the chest demonstrated scattered bilateral pulmonary nodules, many with surrounding ground-glass opacity, along with prominent bilateral axillary lymphadenopathy (Figure 2).

Subsequent workup

Given the newly diagnosed advanced HIV infection, the constellation of right lower extremity edema, violaceous cutaneous lesions, and extensive lymphadenopathy raised concern for cutaneous malignancy versus infection, with possible nodal involvement resulting in lymphatic obstruction and secondary lymphedema. The presence of bilateral pulmonary nodules broadened the differential diagnosis to include disseminated KS, lymphoma, metastatic malignancy, and opportunistic infections, including atypical mycobacterial and fungal infections.

Evaluation for opportunistic infections was pursued. Serum cryptococcal antigen, QuantiFERON TB Gold, and sexually transmitted infection testing were negative. In the setting of a reactive rapid plasma reagin (1:32) and photophobia, ophthalmologic evaluation revealed multiple white dot-appearing chorioretinal lesions, raising concern for ocular syphilis. Based on these findings, ocular syphilis was favored clinically. A subsequent lumbar puncture revealed a mildly elevated opening pressure of 22 cm H_2_O. CSF analysis showed high protein, pleocytosis (predominantly lymphocytes), and a positive CSF VDRL, confirming neurosyphilis. CSF HIV testing was negative (pertinent laboratory studies are summarized in Table 1). Additionally, given persistent odynophagia and dysphagia in the setting of oral candidiasis, esophageal candidiasis was suspected clinically and warranted treatment.

Peripheral blood flow cytometry and serum protein electrophoresis showed no evidence of hematologic malignancy or monoclonal gammopathy. A skin biopsy of the right anterior mid-thigh was performed to establish a definitive diagnosis. Histopathologic analysis confirmed KS, with tumor cells positive for CD31, CD34, ERG, and HHV-8. Subsequently, an excisional biopsy of the right axillary lymph node was performed to evaluate for nodal involvement and exclude alternative lymphoproliferative processes. The results showed spindled cells positive for HHV-8, ERG, CD34, CD31, and D2-40, consistent with disseminated KS.

To further characterize suspected systemic involvement, additional staging studies were pursued. MRI of the brain and orbits was obtained, demonstrating diffuse abnormal signal intensity in the cerebrum, cerebellum, and brainstem (Figure 2). These MRI findings were attributed to the positive syphilis testing on the lumbar puncture. Furthermore, in anticipation of potential future systemic chemotherapy (liposomal doxorubicin) for disseminated or symptomatic disease, transthoracic echocardiography was obtained to assess baseline cardiac function. It showed normal biventricular function and global longitudinal strain, with an ejection fraction of 71%.

Management

Treatment with bictegravir/emtricitabine/tenofovir alafenamide was initiated following the negative serum cryptococcal antigen result. Daily trimethoprim-sulfamethoxazole and fluconazole were started for *Pneumocystis jirovecii* prophylaxis and presumed esophageal candidiasis, respectively. Additionally, intravenous aqueous penicillin G (daily 24 million units for 14 days) was started for the confirmed diagnosis of neurosyphilis.

Although the patient met AIDS Clinical Trials Group (ACTG)-T1 criteria based on tumor‑associated edema and suspected pulmonary involvement, liposomal doxorubicin was deferred until immune reconstitution (CD4 count: >100 cells/µL) could be achieved to avoid further severe immunosuppression. He was scheduled for outpatient follow-up for consideration of the possible timing and necessity of starting systemic chemotherapy.

At the time of this report, the patient remained clinically stable. He had improving thrush symptoms while continuing ART and opportunistic infection prophylaxis. He completed intravenous penicillin, and ocular symptoms ceased. HIV-1 RNA repeat before discharge (eight days after ART initiation) trended down to 5,440 (from 301,000 on admission).

## Discussion

In our patient, several “red flag” clinical features, including violaceous skin lesions over the affected limb, recurrent thrush, generalized lymphadenopathy, and bilateral pulmonary nodules, distinguished the presentation from uncomplicated DVT and pointed toward advanced HIV with KS rather than isolated vascular disease. KS can present initially as unilateral lower extremity edema with violaceous papules or plaques. In this setting, the edema may reflect lymphatic obstruction from nodal disease rather than primary vascular pathology, as reported in the literature on HIV‑associated KS‑related lymphedema [3,4,6]. The negative venous duplex studies and the combination of extensive inguinal/external iliac lymphadenopathy, soft tissue edema, and characteristic cutaneous lesions made a vascular or purely infectious explanation less likely and raised suspicion for KS early in the hospitalization.

This patient’s presentation was strongly consistent with advanced HIV disease, with an AIDS-defining malignancy, KS, persistent oral candidiasis with odynophagia/dysphagia suggesting esophageal involvement, severe CD4 lymphopenia at 72 cells/µL, and a high HIV viral load. Notably, KS is classified by tumor burden as T0 (good risk) or T1 (poor risk) using the AIDS Clinical Trials Group staging system for KS (ACTG) (Table 2) [2]. According to the World Health Organization (WHO) guidelines, immediate ART is recommended for mild to moderate HIV-associated KS [7]. On the other hand, both the WHO and the National Institutes of Health (NIH) recommend combining ART with systemic chemotherapy for severe or symptomatic disease [1,2,7]. For ACTG-T1 disease, NIH guidance specifically lists ART plus liposomal doxorubicin as preferred therapy, which includes tumor-associated edema and visceral involvement (Table 3) [1,2]. Liposomal doxorubicin is considered first-line therapy due to its favorable efficacy and toxicity profile, with response rates exceeding 50%-60% in advanced disease [2,4]. Paclitaxel serves as an effective alternative, particularly in refractory or rapidly progressive cases. Importantly, in patients with a high tumor burden (extensive disease), ART alone may be insufficient, highlighting the need for combined systemic chemotherapy and ART [2,4].

**Table 2:** AIDS Clinical Trials Group Staging System for Kaposi Sarcoma This table illustrates features of both stages of Kaposi sarcoma. T0 indicates good-risk disease. T1 indicates poor-risk disease associated with a higher tumor burden and a need for systemic therapy. In this framework, our patient met ACTG T1 (poor risk) criteria due to tumor-associated edema and suspected visceral involvement. AIDS: acquired immunodeficiency syndrome

Stage	Classification	Criteria
T0	Good risk	Disease confined to skin and/or lymph nodes
		Minimal oral involvement (non-extensive)
		No tumor-associated edema or ulceration
		No visceral involvement
T1	Poor risk	Tumor-associated edema or ulceration
		Extensive oral Kaposi sarcoma
		Visceral involvement (such as pulmonary and gastrointestinal)
		Widespread or rapidly progressive cutaneous disease

**Table 3:** Management Approach to HIV-Associated Kaposi Sarcoma Kaposi sarcoma management recommendations based on disease severity and current NIH/WHO guidance for HIV-associated Kaposi sarcoma. ACTG: AIDS Clinical Trials Group, ART: antiretroviral therapy, HIV: human immunodeficiency virus, NIH: National Institutes of Health, WHO: World Health Organization

Disease severity	Recommended management
Limited disease (ACTG T0)	Initiate ART alone; consider local therapy (radiation and intralesional therapy) if symptomatic
Advanced disease (ACTG T1)	ART plus systemic chemotherapy (liposomal doxorubicin preferred; paclitaxel as an alternative)
Life-threatening or rapidly progressive disease	Urgent systemic chemotherapy in addition to ART is indicated when the disease is associated with extensive visceral involvement or severe symptoms

Pulmonary KS typically presents with bilateral nodules or peribronchovascular infiltrates and may be difficult to distinguish radiographically from opportunistic infections [2,4,8]. In patients with advanced HIV, pulmonary and nodal involvement by KS should be differentiated from opportunistic infections and HHV-8-associated lymphoproliferative disorders, including multicentric Castleman disease [2,4]. In this context, excisional lymph node biopsy is preferred over core sampling to maximize diagnostic yield, allowing comprehensive histopathologic, flow cytometric, microbiologic, and HHV-8-directed evaluation [2,7]. In our case, the right inguinal lymph node was more accessible for evaluation of lymph node involvement, so a pulmonary biopsy was deferred.

Furthermore, our case underscores the importance of not anchoring on a single diagnosis in advanced HIV with possible opportunistic infections (Table 4). Although KS explained the unilateral edema and cutaneous lesions [5,9], the patient simultaneously had presumed esophageal candidiasis and neuro- and ocular syphilis. CDC guidance states that patients with ocular symptoms and reactive syphilis serology require urgent ophthalmologic assessment [5]. If ocular syphilis is suspected, treatment should proceed as for neurosyphilis with aqueous crystalline penicillin G 18-24 million units daily for 10-14 days, even when cerebrospinal fluid findings are normal [5]. In our case, bilateral white dot chorioretinal lesions in the setting of reactive serologies and uncertain prior treatment justified empiric IV penicillin, even before obtaining the CSF-positive VDRL results. This case also reflects a common modern pattern: late HIV diagnosis despite prior clinical warning signs. The patient had recurrent thrush documented months earlier and limited access to ongoing care. Persistent or recurrent oral candidiasis in an otherwise young adult, especially when accompanied by unexplained hypergammaglobulinemia, lymphadenopathy, or constitutional symptoms, should prompt HIV testing and urgent linkage to care [3,9].

**Table 4:** Common Opportunistic Infections in Advanced HIV by CD4 Count CD4: cluster of differentiation 4, cells/µL: cells per microliter, PJP: *Pneumocystis jirovecii* pneumonia, CNS: central nervous system, CMV: cytomegalovirus, MAC: mycobacterium avium complex

CD4 count (cells/µL)	Common opportunistic infections	Key clinical features
<500	Oral candidiasis, herpes zoster, tuberculosis	Thrush, dermatomal rash, chronic cough
<200	PJP, esophageal candidiasis	Dyspnea, hypoxia, odynophagia/dysphagia
<100	Toxoplasmosis, cryptococcosis	CNS lesions, meningitis symptoms
<50	CMV, MAC	Retinitis, systemic illness, weight loss

Finally, this case highlights an important management nuance regarding ART timing and immune reconstitution inflammatory syndrome (IRIS) [2,10]. Although IRIS can occur after ART initiation in KS, early ART initiation is recommended, as the benefits generally outweigh the risk. ART should not be delayed unless a competing central nervous system opportunistic infection, such as cryptococcal meningitis, is suspected or confirmed [2]. In this patient, a negative serum cryptococcal antigen supported prompt ART initiation. Current WHO and NIH guidelines similarly recommend early ART, with the addition of systemic therapy in patients with advanced disease [1,2,7].

## Conclusions

This case highlights KS as an initial manifestation of previously undiagnosed advanced HIV infection, presenting atypically as unilateral lower extremity lymphedema with cutaneous lesions. Biopsy confirmation of cutaneous and nodal KS, together with imaging evidence of widespread lymphadenopathy and pulmonary nodules, demonstrated disseminated disease in this patient. It underscores the importance of maintaining a broad differential diagnosis in immunocompromised patients, particularly when vascular studies are unrevealing. The coexistence of opportunistic and sexually transmitted infections further emphasizes the need for comprehensive evaluation at the time of HIV diagnosis. Prompt recognition, early tissue confirmation, timely initiation of ART, and appropriate systemic management, illustrated here by the patient’s early symptomatic improvement and virologic response, are critical to optimizing outcomes in patients with advanced HIV and suspected disseminated disease.
